# A metal-free electrocatalyst for carbon dioxide reduction to multi-carbon hydrocarbons and oxygenates

**DOI:** 10.1038/ncomms13869

**Published:** 2016-12-13

**Authors:** Jingjie Wu, Sichao Ma, Jing Sun, Jake I. Gold, ChandraSekhar Tiwary, Byoungsu Kim, Lingyang Zhu, Nitin Chopra, Ihab N. Odeh, Robert Vajtai, Aaron Z. Yu, Raymond Luo, Jun Lou, Guqiao Ding, Paul J. A. Kenis, Pulickel M. Ajayan

**Affiliations:** 1 Department of Materials Science and NanoEngineering, Rice University, 6100 Main Street, Houston, Texas 77005, USA; 2 School of Chemical Sciences, University of Illinois at Urbana-Champaign, Urbana, Illinois 61801, USA; 3 International Institute for Carbon-Neutral Energy Research (WPI-I2CNER), Kyushu University, 744 Moto-oka, Nishi-ku, Fukuoka 819-0395, Japan; 4 State Key Laboratory of Functional Materials for Informatics, Shanghai Institute of Microsystem and Information Technology, Chinese Academy of Sciences, Shanghai 200050, China; 5 Saudi Basic Industries Corporation (SABIC), Sugar Land, Texas 77478, USA

## Abstract

Electroreduction of carbon dioxide into higher-energy liquid fuels and chemicals is a promising but challenging renewable energy conversion technology. Among the electrocatalysts screened so far for carbon dioxide reduction, which includes metals, alloys, organometallics, layered materials and carbon nanostructures, only copper exhibits selectivity towards formation of hydrocarbons and multi-carbon oxygenates at fairly high efficiencies, whereas most others favour production of carbon monoxide or formate. Here we report that nanometre-size N-doped graphene quantum dots (NGQDs) catalyse the electrochemical reduction of carbon dioxide into multi-carbon hydrocarbons and oxygenates at high Faradaic efficiencies, high current densities and low overpotentials. The NGQDs show a high total Faradaic efficiency of carbon dioxide reduction of up to 90%, with selectivity for ethylene and ethanol conversions reaching 45%. The C2 and C3 product distribution and production rate for NGQD-catalysed carbon dioxide reduction is comparable to those obtained with copper nanoparticle-based electrocatalysts.

## Abstract (web summary)

Electroreduction of CO_2_ into C2 hydrocarbons and liquid fuels is a promising but challenging energy conversion technology, with copper exhibiting fair selectivity for these products. Here, the authors report that N-doped graphene quantum dots can also catalyze the electrochemical reduction of CO_2_ into multi-carbon hydrocarbons and oxygenates.

Electroreduction of carbon dioxide (CO_2_) with protons from water into valued commodity chemicals offers a potential route towards a carbon-neutral society^1^. However, the development of this technology is hampered by the lack of active catalysts converting CO_2_ into more-energy embedded fuels or useful chemicals. Most metallic catalysts prefer the competitive off-pathway reduction of water to hydrogen, whereas others such as Au, Ag, Pb and Sn primarily catalyse production of carbon monoxide (CO) or formate (HCOO^−^) via a two-electron transfer pathway^2^,^3^. On the other hand, organometallic, two-dimensional materials and nitrogen (N)-doped nanostructured carbon materials in the form of few layer graphene, carbon nanotubes (CNTs) and carbon fibres have been found only capable of catalysing CO_2_ reduction into CO^4^,^5^,^6^,^7^,^8^. To date, among the huge amount of screened materials, only Cu or copper oxide-derived catalyst are known to drive the electrolysis of CO_2_ into low-carbon hydrocarbons and oxygenates at fairly high Faradaic efficiencies (FEs) through a multiple-step electron transfer pathway^9^,^10^,^11^. However, the overpotentials for hydrocarbons and oxygenates production are usually high (−0.7 V) on most Cu catalysts, whereas the overall current densities are typically below 50 mA cm^−2^ (refs 2, 11, 12, 13). Therefore, more efforts have to be devoted, to develop more powerful catalysts for efficient CO_2_ conversion to multi-carbon hydrocarbons and oxygenates. Reducing the structural features to sub-nanometre dimensions often significantly enhances the hydrogenation activity for metal catalysts and it even has been demonstrated to transform a non-catalytic active bulk counterpart into a highly active catalyst towards CO_2_ reduction^14^,^15^,^16^,^17^. Separately, the introduction of additional defects in carbon nanostructures by heteroatom doping gives rise to activity toward CO_2_ activation^6^,^7^,^8^,^18^,^19^.

Here we combine a high inherent defect density, achieved by tuning the dimensions and morphology of carbon nanostructure into nanometre scale, with foreign N-doping to obtain an advanced metal-free catalyst for electroreduction of CO_2_ to value-added chemicals. The resulting catalyst, specifically N-doped graphene quantum dots (NGQDs), has substantially enriched density of N-doping defects at edge sites. These NGQDs exhibit high activity towards the electrochemical reduction of CO_2_ as evidenced by high reduction current densities at low overpotentials and, more importantly, they preferentially produce multi-carbon hydrocarbons and oxygenates, especially the C2 products ethylene (C_2_H_4_) and ethanol (C_2_H_5_OH) at FEs comparable to those obtained using Cu nanoparticle-based catalysts.

## Results

### NGQD synthesis

**Figure 1:**
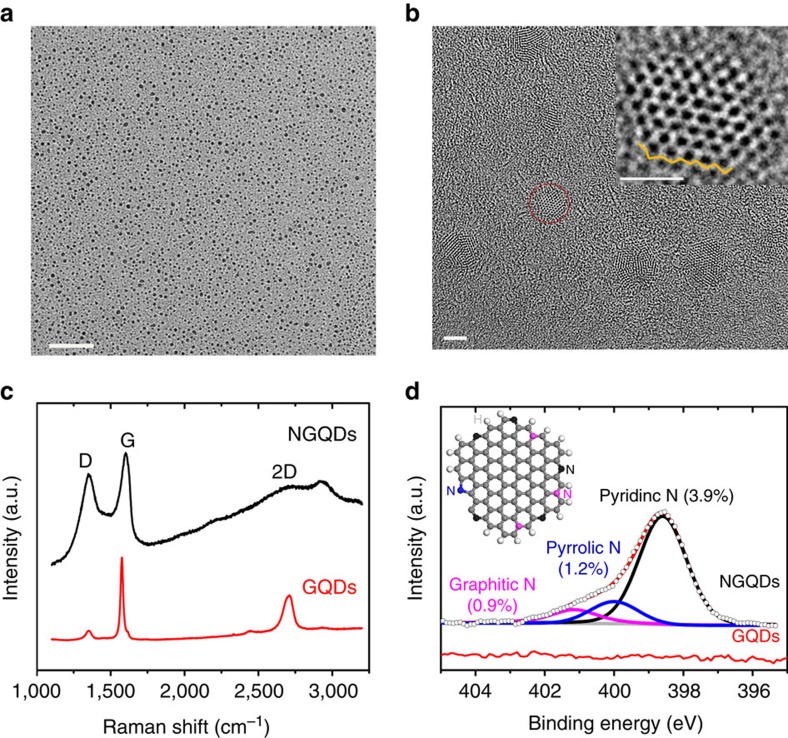
N**anostructure and specific nitrogen dopant configurations of NGQDs**. (**a**) Low-magnification TEM image of NGQDs. Scale bar, 50 nm. (**b**) High-magnification TEM image of NGQDs. Scale bar, 2 nm. Inset shows a single NGQD containing zigzag edges as circled. The yellow line outlines the zigzag edge. Scale bar in inset, 1 nm. (**c**) Raman spectrum of NGQDs as compared with that of pristine GQDs. (**d**) High-resolution N 1*s* spectrum for NGQDs, deconvoluted into three sub-peaks representing pyridinic, pyrrolic and graphitic N. The value in parentheses is the corresponding N atomic concentration calculated based on N/(N+C). The inset is a schematic demonstrating zigzag edges and the N-bonding configuration with respect to pyridinic (black), pyrrolic (blue) and graphitic (pink) N. In comparison, the N 1*s* peak does not appear in GQDs sample.

To expose the edge sites and increase their density in carbon nanostructures while simultaneously introducing non-metal heteroatom dopants at the edge locations, we intended to synthesize NGQDs through exfoliating and cutting graphene oxide (GO) precursor, and *in-situ* N doping in the dimethylformamide (DMF) solvent at elevated temperature and pressure (for synthesis details, see Supplementary Methods)^20^. The obtained NGQDs have a thickness between 0.7 and 1.8 nm, corresponding to one to three atomic layers (Supplementary Fig. 1), and a narrow lateral size distribution of 1–3 nm (Fig. 1a,b and Supplementary Fig. 1). The high-resolution transmission electron microscopy (TEM) images show the hexagonal-like morphology of NGQDs and their honeycomb framework (inset in Fig. 1b showing a representative zigzag edge), indicating the preservation of the graphene crystal structure. High-resolution TEM images of NGQDs with distinguishable atoms are difficult to take, as the NGQDs are even thinner than the carbon support film in the TEM grid. We do not have a large quantity sample base to analyse the percentage of NGQDs with zigzag edges over armchair edges, but the exigence of NGQDs with armchair edges could not be excluded in our sample. Pristine GQDs, without N-doping, were also synthesized from the same GO precursor using the identical hydrothermal analogous treatment in a solvent mixture of IPA/H_2_O (1:1 by volume) instead of DMF^21^. The pristine GQDs exhibit a similar morphology (thickness and lateral size) as the NGQDs (Supplementary Fig. 2). The Raman spectrum of NGQDs shows the characteristic D band at 1,350 cm^−1^ and G band at 1,589 cm^−1^, whereas the second order band (2D) has been quenched compared with pristine GQDs (Fig. 1c). The ratio of *I*_D_/*I*_G_ (D band intensity to G band intensity) for NGQDs is much higher than that for the pristine GQDs, due largely to the introduction of additional defects on N doping. The survey scan of X-ray photoelectron spectroscopy (XPS) clearly shows the presence of N in the NGQDs, whereas the N 1*s* peak was not observed for pristine GQDs (Supplementary Fig. 3). Analysis of the fine scan of the N 1*s* region provides information on the specific configuration of the N dopants in the carbon lattice network (Fig. 1d). Deconvolution of the N 1*s* peak reveals three peaks that can be assigned to pyridinic (398.5 eV), pyrrolic (400.0 eV) and graphitic N (401.2 eV)^22^. The total N content in NGQDs based on N/(C+N) is around 6.0 at.%, in which pyridinic N is predominant with ∼3.9 at.% (Fig. 1d and Supplementary Fig. 3).

### Electrocatalytic activity and selectivity towards CO_2_ reduction

**Figure 2:**
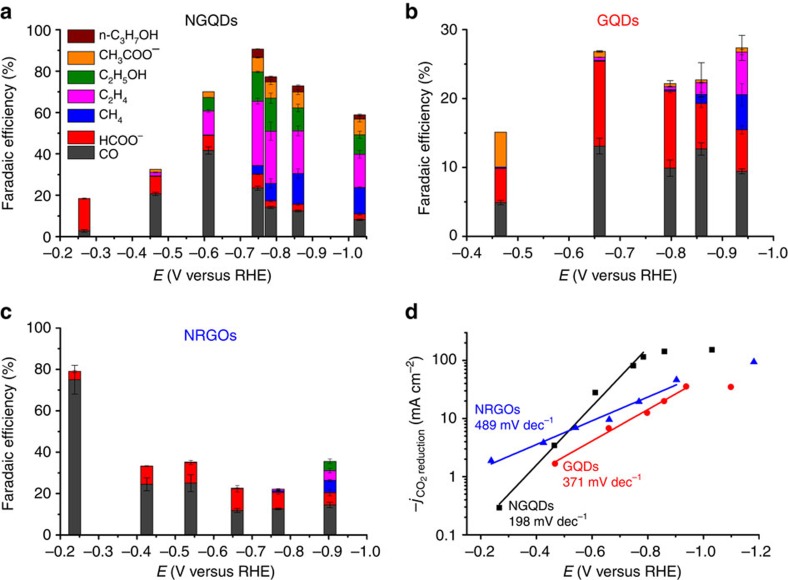
Electrocatalytic activity of carbon nanostructures towards CO_2_ reduction. (**a**) FEs of carbon monoxide (CO), methane (CH_4_), ethylene (C_2_H_4_), formate (HCOO^−^), ethanol (EtOH), acetate (AcO^−^) and n-propanol (n-PrOH) at various applied cathodic potential for NGQDs. (**b**) FE of CO_2_ reduction products for pristine GQDs. (**c**) Selectivity to CO_2_ reduction products for NRGOs. (**d**) Tafel plots of partial current density of CO_2_ reduction versus applied cathodic potential for three nanostructured carbon catalysts. The error bar represents the s.d. of three separate measurements for an electrode.

The electrocatalytic activity and selectivity of NGQDs towards CO_2_ reduction were evaluated in a flow cell in which NGQDs were deposited on a gas diffusion electrode (Supplementary Fig. 4)^9^. The electrolysis was performed in an electrolyte of 1 M KOH in the potentiostatic mode with cathodic potentials in the range of −0.20 to −1.10 V (versus reversible hydrogen electrode (RHE)). The total FE of CO_2_ reduction increases as a more negative potential is applied, from 18% at −0.26 V to a maximum of 90% at −0.75 V and, subsequently, it declines to 64% at −1.03 V (Fig. 2a). Only CO and HCOO^−^ are observed at a low cathode potential of −0.26 V. However, when the applied potential sweeps more negatively, various hydrocarbons of methane (CH_4_) and C_2_H_4_, and multi-carbon oxygenates including C_2_H_5_OH, acetate (CH_3_COO^−^) and *n*-propanol (*n*-C_3_H_7_OH) are produced in addition to CO and HCOO^−^ (Fig. 2a). The fact that NGQDs favour catalysing C–C bond formation beyond the potential of −0.61 V, leading to predominant production of C2 and C3 products, is striking (Fig. 2a and Supplementary Fig. 5). C_2_H_4_ is the major hydrocarbon product with a maximum FE of 31% at −0.75 V, whereas CH_4_ has a maximum FE of only 15% at −0.86 V. In addition to C2 hydrocarbons, another prominent feature of the NGQDs lies in its outstanding selectivity to formation of multi-carbon oxygenate liquid fuels. At −0.78 V, the maximum FE for multi-carbon oxygenates reaches 26%, with the major component C_2_H_5_OH accounting for 16%. In comparison, Cu nanoparticle-based catalysts yield trace CH_4_ (<1%), but comparable C_2_H_4_ (31.2%) and C_2_H_5_OH (11.8%) at the similar potential of −0.74 V (Supplementary Fig. 6)^9^. Overall, the NGQD catalyst exhibits electrocatalytic performance towards CO_2_ reduction that is similar to what has been observed for Cu nanoparticle catalysts in terms of the FE for C2 and C3 products.

To verify that the CO_2_ reduction reaction was catalysed by NGQDs, multiple control experiments were conducted. In one control experiment, argon gas was supplied to the cathode compartment instead of CO_2_, which resulted in the exclusive production of hydrogen over the cathodic potential window of −0.2 ∼ −1.0 V. In a second control experiment using a bare gas diffusion electrode as the cathode (no NGQD catalyst), hydrogen formation prevailed over the same potential region. Furthermore, in an experiment using an isotopically labelled ^13^CO_2_ gas feed, mass spectrometry showed signals for ^13^CO (*m*/*z*=29), ^13^CH_4_ (*m*/*z*=17) and ^13^C_2_H_4_ (*m*/*z*=30), whereas a spin doublet appeared in ^1^H NMR due to proton coupling to ^13^C in the liquid products (Supplementary Fig. 7), all confirming that these products are derived from ^13^CO_2_ electroreduction. In addition, inductively coupled plasma optical emission spectroscopy revealed that the NGQDs samples only contained 1.02 p.p.m. Cu, corresponding to a Cu loading of around 5 × 10^−3^ μg cm^−2^ on the cathode, which is too low to catalyse CO_2_ reduction to hydrocarbons and oxygenates at the levels observed here, as previously reported^23^. These results confirm the activity of the NGQDs studied here towards efficient reduction of CO_2_ to multi-carbon hydrocarbons and oxygenates.

### Origin of electrocatalytic activity and selectivity

To unveil the origin of the activity and selectivity of NGQDs, pristine GQDs were also tested for CO_2_ reduction under identical experimental conditions. The hydrogen evolution reaction dominates over the CO_2_ reduction reaction for the GQDs electrode. GQDs not only initialize CO_2_ reduction at a more negative potential, but also primarily produce CO and HCOO^−^ at even lower FEs than NGQDs (Fig. 2b). Compared with NGQDs, the FEs of hydrocarbons such as CH_4_ and C_2_H_4_ observed with the GQDs are low not more than 5–6% each. Moreover, only a trace amount of CH_3_COO^−^ and no C_2_H_5_OH or *n*-C_3_H_7_OH were observed. In addition, the Tafel plots derived from the partial current density of CO_2_ reduction (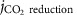) versus cathode potential show that the GQDs electrode has a larger apparent Tafel slope than the NGQDs electrode (371 mV dec^−1^ for GQDs versus 198 mV dec^−1^ for NGQDs), indicating that the GQDs electrode exhibits much slower kinetics than the NGQDs electrode (Fig. 2d). This NGQDs versus GQDs performance comparison underscores the significance of the N-doping defects in the NGQD catalyst in determining its activity. Previously, the incorporation of N defects to *sp*^2^ carbon nanostructures to induce charge density was reported to be crucial for actively catalysing electrochemical reactions such as oxygen reduction reaction (ORR)^24^,^25^,^26^. Among the most common N configurations, pyridinic N is experimentally proven to create the most active site for the ORR^27^,^28^,^29^. Similar to ORR, our prior work on N-doped CNTs and graphene suggests that pyridinic N is the leading active site for CO_2_ reduction^6^,^7^. The acidic CO_2_ molecule prefers adsorbing onto the Lewis basic pyridinic N group in carbon nanostructures^27^.

The NGQDs samples were analysed by *ex-situ* post-reaction XPS. After testing for CO_2_ reduction, the relative concentration of pyridinic N (398.5 eV) decreases from 65 to 38% and another N component with a peak of 400.0 eV (pyrrolic or pyridonic N) increases from 20 to 50%, whereas the percentage of graphitic N (401.2 eV) remains relatively unchanged (from 15 to 12%; Supplementary Fig. 8). The change of N component fraction before and after CO_2_ reduction measurement possibly results from the adsorption of CO_2_ onto the active pyridinic N that causes the upshift of binding energy of pyridinic N to a similar value of pyrrolic N. By applying potential, the adsorbed CO_2_ is further reduced to products that are released from the pyridinic N site, then the pyridinic N regenerates for next round of CO_2_ adsorption/reduction.

To further explore the active site, the N-doped reduced GOs (NRGOs) were prepared by doping GOs with NH_3_ at 800 °C. The NRGOs contain a concentration of each specific N configuration that is similar to the distribution in NGQDs, but the NRGOs have larger micrometre-scale lateral size (Supplementary Figs 9 and 10). The onset potential for CO_2_ reduction on the NRGOs electrode is identical to that for the NGQDs electrode (Fig. 2a,c). However, the NRGOs electrode primarily catalyses CO_2_ reduction to form CO (Fig. 2c), similar to previously reported observations when using N-doped graphene and N-doped CNTs^6^,^7^. The formation of noticeable amounts of hydrocarbons CH_4_ (∼6%), C_2_H_4_ (∼5%) and oxygenates C_2_H_5_OH (∼4%) starts at −0.90 V on NRGOs, a significantly more negative potential than that (−0.61 V) on NGQDs. Compared with NRGOs, pristine RGOs exclusively catalyse the hydrogen evolution reaction, underlining the ability of N doping to transform carbon nanostructures from being inert to highly catalytically active. The kinetics for CO_2_ reduction on NRGOs is also slower than that on NGQDs, for example, a Tafel slope of 489 mV dec^−1^ for NRGOs versus 198 mV dec^−1^ for NGQDs (Fig. 2d). This comparison of NGQDs and NRGOs clearly suggests that the morphology also plays a key role in determining the activity of carbon nanostructures towards CO_2_ reduction in addition to doping-induced defects. When 1–3 μm diameter NRGOs sheets are pulverized into 1–3 nm quantum dots, the density of exposed edge sites increases by three orders of magnitude: 0.05% for 1 μm NRGO sheet versus 44% for 1 nm NGQD (Supplementary Fig. 11). Owing to the lower formation energy of N doping at the edge sites than the basal planes, especially for pyridinic N, most of the N dopants would locate at the edge sites in NGQDs^30^,^31^. In contrast, in the case of NRGOs, N dopants mostly locate in the basal planes because insufficient edge sites are present to accommodate the dopants. Although they are similar in pyridinic N content, NGQDs tend to have higher density of pyridinic N at edge sites than NRGOs. The pyridinic N at the edge site is believed to be more active to induce C–C bond formation than those at the basal plane, which leads to a higher yield of C2 and C3 products on NGQDs electrode than on NRGOs electrode^32^.

### Production rate of CO_2_ reduction products

**Figure 3:**
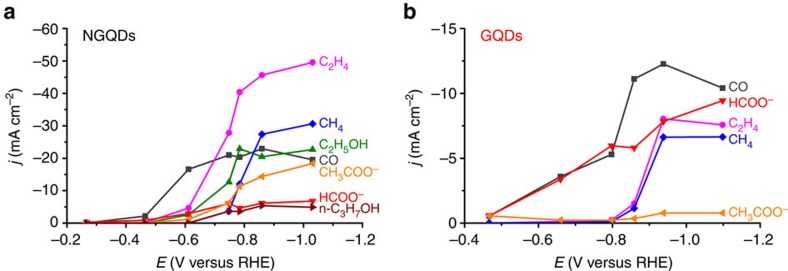
Partial current densities as a function of cathode potential for various products from electrochemical CO_2_ reduction. (**a**) When using NGQDs as cathode catalyst. (**b**) When using GQDs as cathode catalyst.

In addition to the promising selectivity with respect to the formation of multi-carbon hydrocarbons and oxygenates, the NGQDs electrode also exhibits a high CO_2_ reduction current density of 100 mA cm^−2^ at relative low potentials (Supplementary Fig. 12). The partial current densities for production of CO, C_2_H_4_ and C_2_H_5_OH reach 23, 46 and 21 mA cm^−2^ at −0.86 V, respectively (Fig. 3a), which are on the same order of magnitude compared with corresponding partial current densities observed for commercial Cu nanoparticles (around 20–40 nm) under identical testing conditions^9^. Accordingly, CO, C_2_H_4_ and C_2_H_5_OH could be produced at rates of 4.2, 1.4 and 0.7 mol h^−1^ m^−2^ at −0.86 V, respectively (Supplementary Fig. 13). In contrast, the GQDs exhibit a partial current density or production rate for C_2_H_4_ that is at least one order of magnitude lower at comparable potentials (Fig. 3b and Supplementary Fig. 13).

## Discussion

A metal-free catalyst comprising NGQDs is discovered to exhibit extraordinary activity towards CO_2_ reduction. Interestingly, NGQDs show predominant selectivity to the production of multi-carbon hydrocarbons and oxygenates, whereas GQDs and NRGOs primarily yield CO and HCOO^−^. Detailed characterization reveals that the unique nanostructure in combination with utmost exposure of edge sites and heteroatom N doping grants NGQDs the unprecedented activity and selectivity. This finding expands the horizon for the design of high-performance electrocatalysts for CO_2_ reduction. However, the reaction mechanism of electroreduction of CO_2_ over NGQDs remains elusive and the unravelling requires further work. The combination of high-sensitive operando spectroscopy and first-principle calculation would be an efficient way to provide insight into the reaction intermediates to reveal the reaction pathways. In addition, the activity difference between the zig-zag and armchair edge necessitates exploration as well in the future.

## Methods

### GQD synthesis

NGQDs were synthesized by heat treating the GO dispersion in DMF in a PTFE-lined autoclave at 200 °C for 10 h. The pristine GQDs were prepared in a similar way, except using a mixture of isopropanol and water (1:1 by volume) as the solvent. The NRGO was prepared by doping GO in a tube furnace at 800 °C, while flowing ammonia for 1 h. More details about the synthesis and characterization are in the Supplementary Information.

### Materials characterization

The morphology and crystallinity of N-doped and pristine GQDs was characterized by high-resolution field-emission gun TEM (JEOL 2,100 FEG TEM). The TEM samples were prepared by dropping the QDs solution onto the ultrathin carbon film TEM grid followed by vacuum drying at 100 °C. The thickness of QDs was measured by atomic force microscopy with tapping mode (Bruker Multimode 8). The atomic force microscopy samples were prepared by dropping the QDs solution onto the Mica substrate. The morphology of NRGOs was analysed by scanning electron microscope (FEI Quanta 400 FEG ESEM). The Raman spectra were taken with a Renishaw inVia Raman microscope with 514 nm laser excitation. XPS measurements were performed to analyse the element component and oxide state of QDs and NRGOs at ambient temperature using PHI Quantera with Al-Kα X-ray source. The Cu content in the NGQDs was determined by using inductively coupled plasma optical emission spectroscopy (PerkinElmer-Optima 2000DV). One millilitre of original liquid sample that is dissolved in DMF was diluted with 5 ml water, then one drop (∼0.05 ml) of concentrated HNO_3_ was used to adjust the pH to be <7. Two different emission lines: *λ*=224.7000, nm and *λ*=213.5970, nm were used to detect the Cu element. The detection limit for Cu is 10 p.p.b.

### Electrochemical measurement of CO_2_ reduction

The CO_2_ reduction reaction was conducted in an electrochemical flow cell composed of targeted quantum dots or NRGO-based gas diffusion electrodes (see Supplementary Fig. 4). The electrochemical measurement was carried out at ambient pressure and temperature. The electrolysis was performed under potentiostatic mode with a full cell voltage ranging from −1.6 to −3.5 V controlled by a potentiostat (Autolab PGSTAT-30, EcoChemie). Both the catholyte and anolyte were 1 M KOH (pH 13.48, as calibrated by a pH meter (Thermo Orion, 9106BNWP). Individual electrode potentials were recorded using multimeters (AMPROBE 15XP-B) connected to each electrode and a reference electrode (Ag/AgCl; RE-5B, BASi) placed in the electrolyte exit stream. The measured potentials after iR compensation were rescaled to the RHE by E (versus RHE)=E (versus Ag/AgCl)+0.209 V+0.0591 V/pH × pH. The current reported here was obtained by averaging the span of time (at least 180 s) for each applied voltage.

For each applied voltage, after the cell reached steady state, 1 ml of the effluent gas stream was periodically sampled and diverted into a gas chromatograph (Thermo Finnegan Trace GC) equipped with both the thermal conductivity and flame ionization detector, and a Carboxen 1,000 column (Supelco). Meanwhile, the exit catholyte was collected at each applied voltage followed by identifying and quantifying using ^1^H NMR (nuclear magnetic resonance, UI500NB, Varian).

### Data availability

The data that support the findings of this study are available from the corresponding author upon request.

## Additional information

**How to cite this article:** Wu, J. *et al*. A metal-free electrocatalyst for carbon dioxide reduction to multi-carbon hydrocarbons and oxygenates. *Nat. Commun.* **7,** 13869 doi: 10.1038/ncomms13869 (2016).

**Publisher's note:** Springer Nature remains neutral with regard to jurisdictional claims in published maps and institutional affiliations.

## Supplementary Material

Supplementary Information Supplementary Figures, Supplementary Methods and Supplementary References.
