# Supplementary material for: A metal-free electrocatalyst for carbon dioxide reduction to multi-carbon hydrocarbons and oxygenates
Source: Nat Commun. 2016 Dec 13;7:13869. doi: 10.1038/ncomms13869 (PMC5159826; doi:10.1038/ncomms13869)
Supplement: Supplementary Information — Supplementary Figures, Supplementary Methods and Supplementary References. [file ncomms13869-s1.pdf]

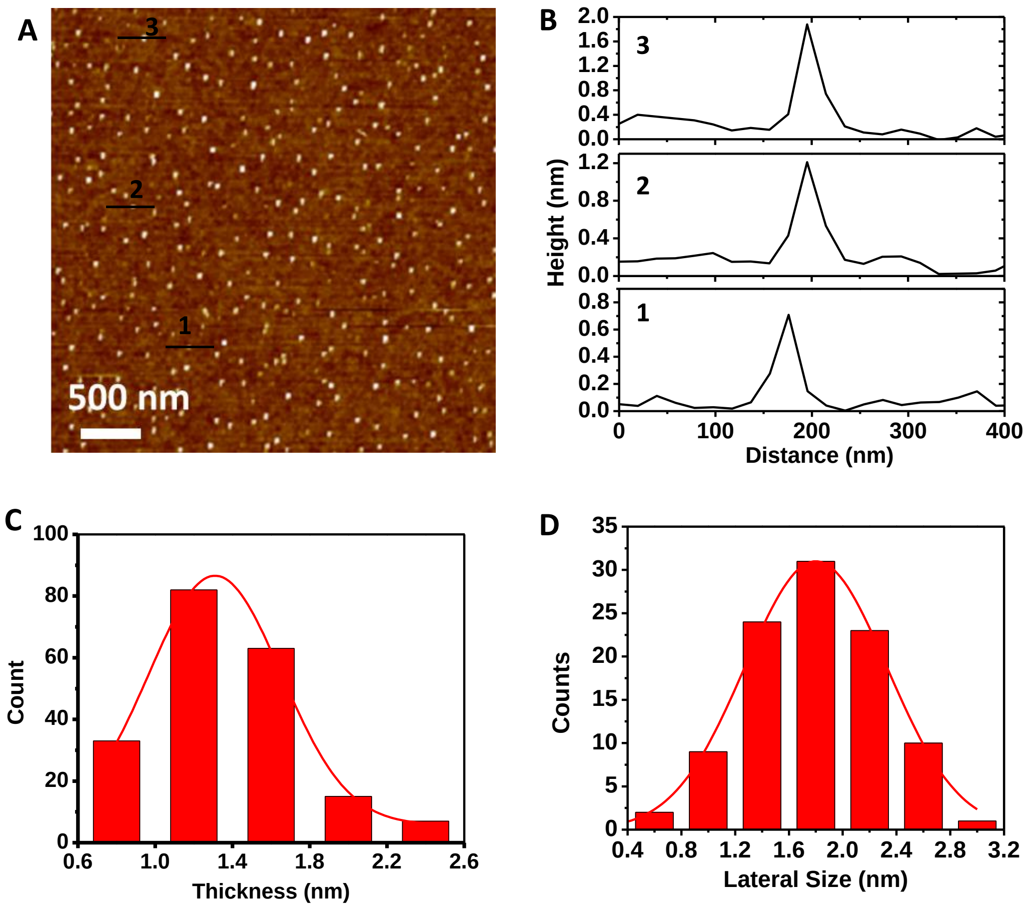

Supplementary Figure 1. Morphology and structure of NGQDs. (A) AFM image of NGQDs. (B) Thickness profiles corresponding to the label in (A). (C) Statistical thickness. (D) Statistical lateral size.

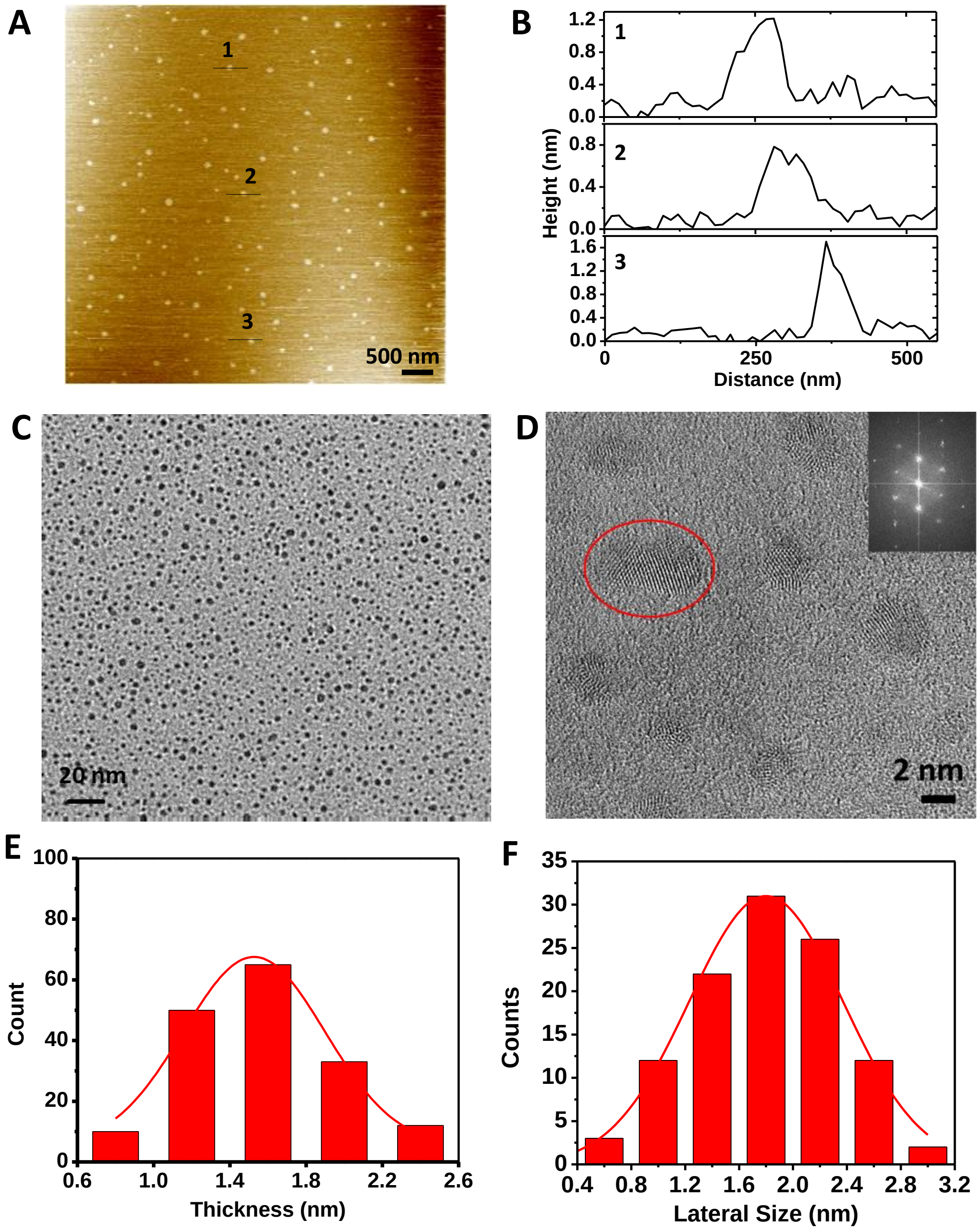

Supplementary Figure 2. Morphology and structure of pristine GQDs. (A) AFM image of pristine GQDs. (B) Thickness profiles corresponding to the label in (A). (C) Low-magnification TEM images of GQDs. (D) High-magnification TEM images. The inset is the fast Fourier transform pattern of a circled single GQD. (E) Statistical thickness. (F) Statistical lateral size.

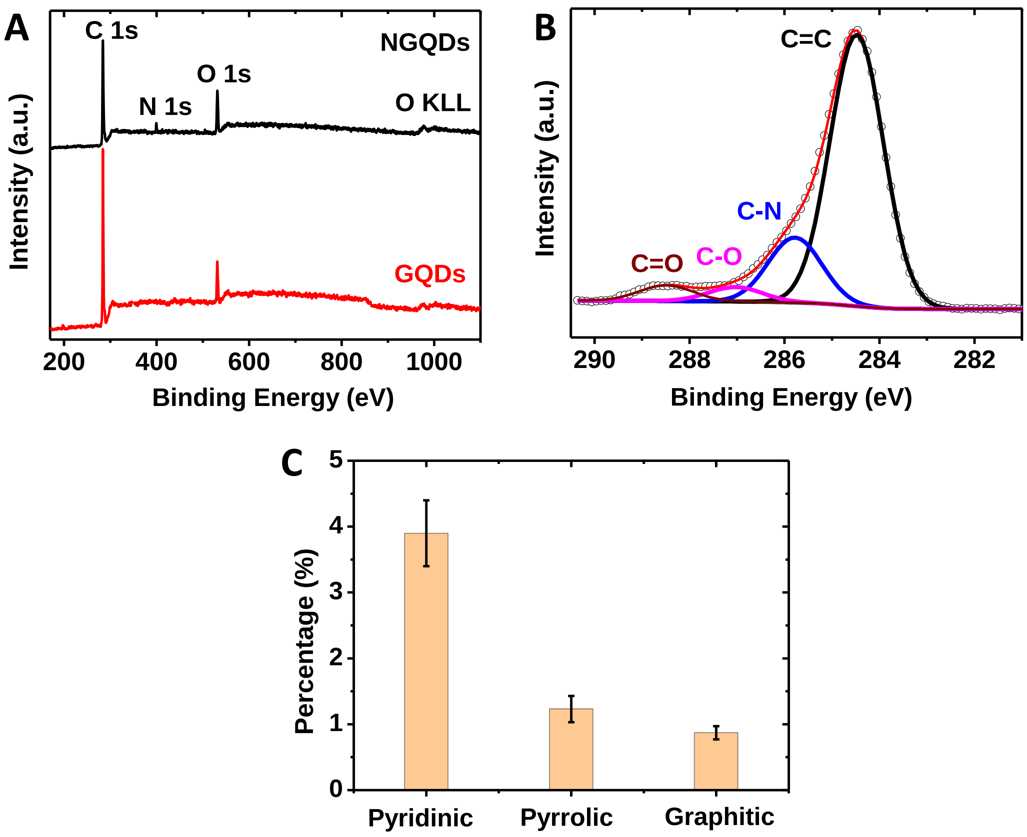

Supplementary Figure 3. XPS analysis of NGQDs and GQDs. (A) Survey scan of XPS for NGQDs and GQDs. (B) C1s peak for NGQDs showing a C-N fitting peak. (C) The concentrations of specific N configurations in NGQDs calculated according to N/(C+N).

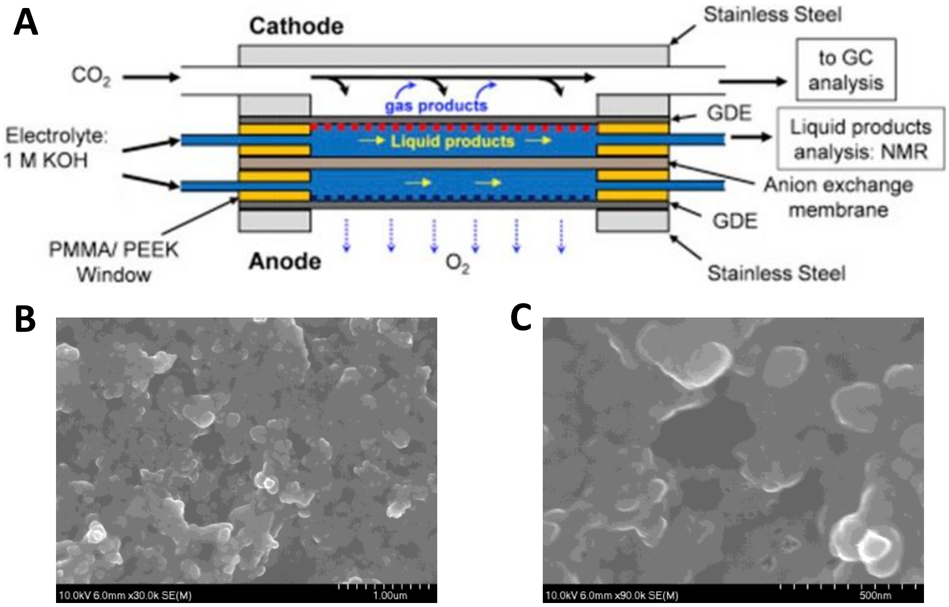

Supplementary Figure 4. Reactor and electrode for electrochemical CO_2 reduction. (A) Schematic of flow cell for CO_2 reduction. (B) Low-magnification SEM image of a NGQDs gas diffusion electrode. (C) High-magnification SEM image of a NGQDs gas diffusion electrode.

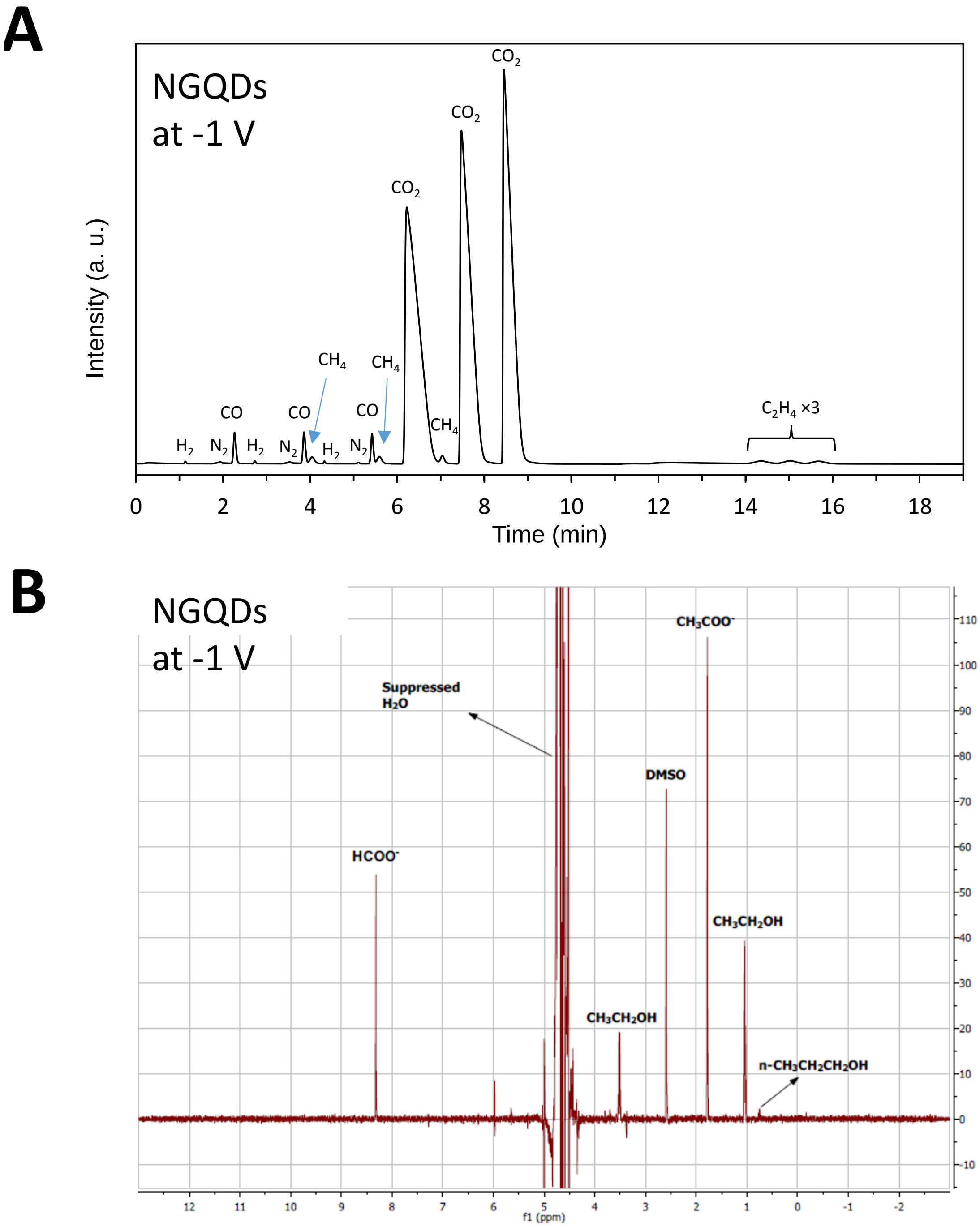

Supplementary Figure 5. Products detection. (A) GC trace of the gas products. (B) NMR spectrum of liquid products for one run at around -1.0 V vs. RHE for the NGQDs electrode.

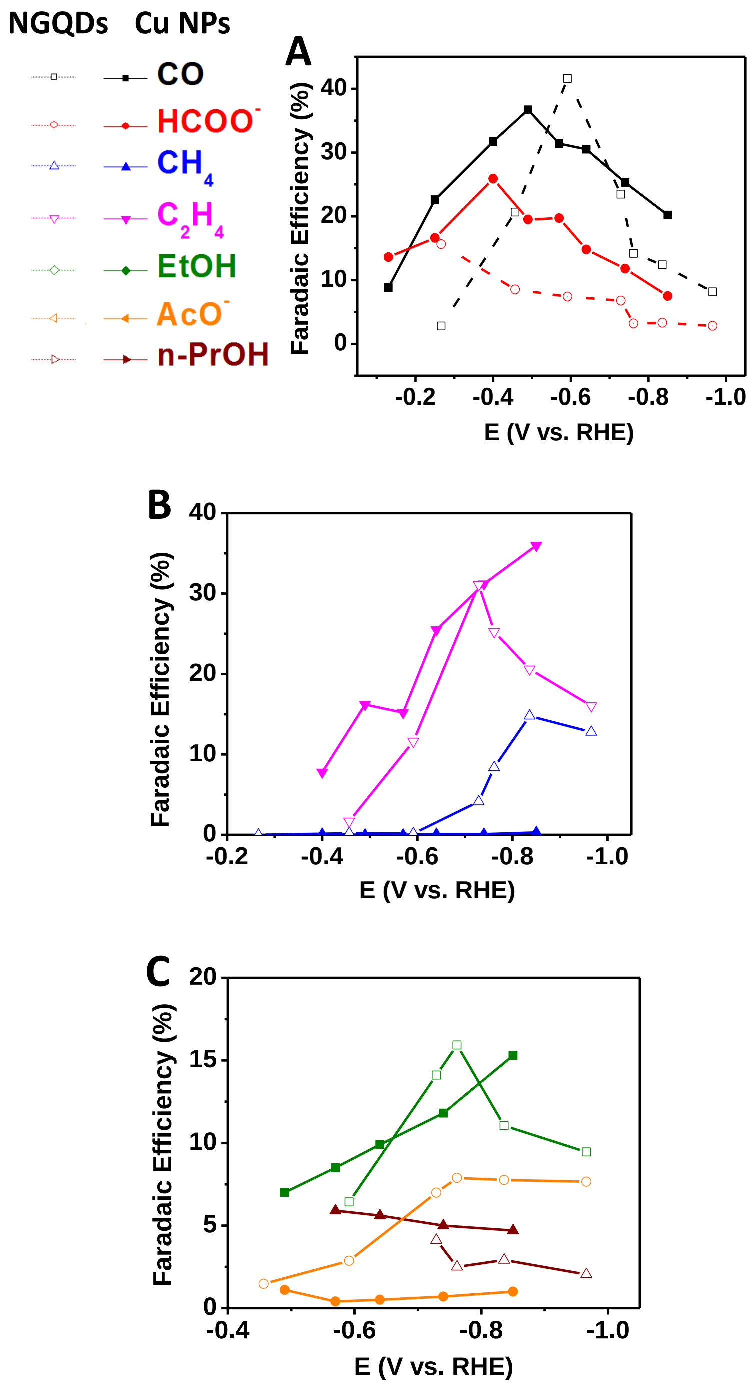

Supplementary Figure 6. Comparison of Faradaic efficiencies of products between NGQDs and commercial Cu nanoparticles (20-40 nm). (A) Carbon monoxide (CO) and formate ($HCOO^-$). (B) Hydrocarbons of methane (CH_4) and ethylene (C_2H_4). (C) Multi-carbon oxygenates of ethanol (C_2H_5OH), acetate (AcO^-), and n-isopropanol (n-PrOH). The Cu nanoparticles data were adopted from reference (5).

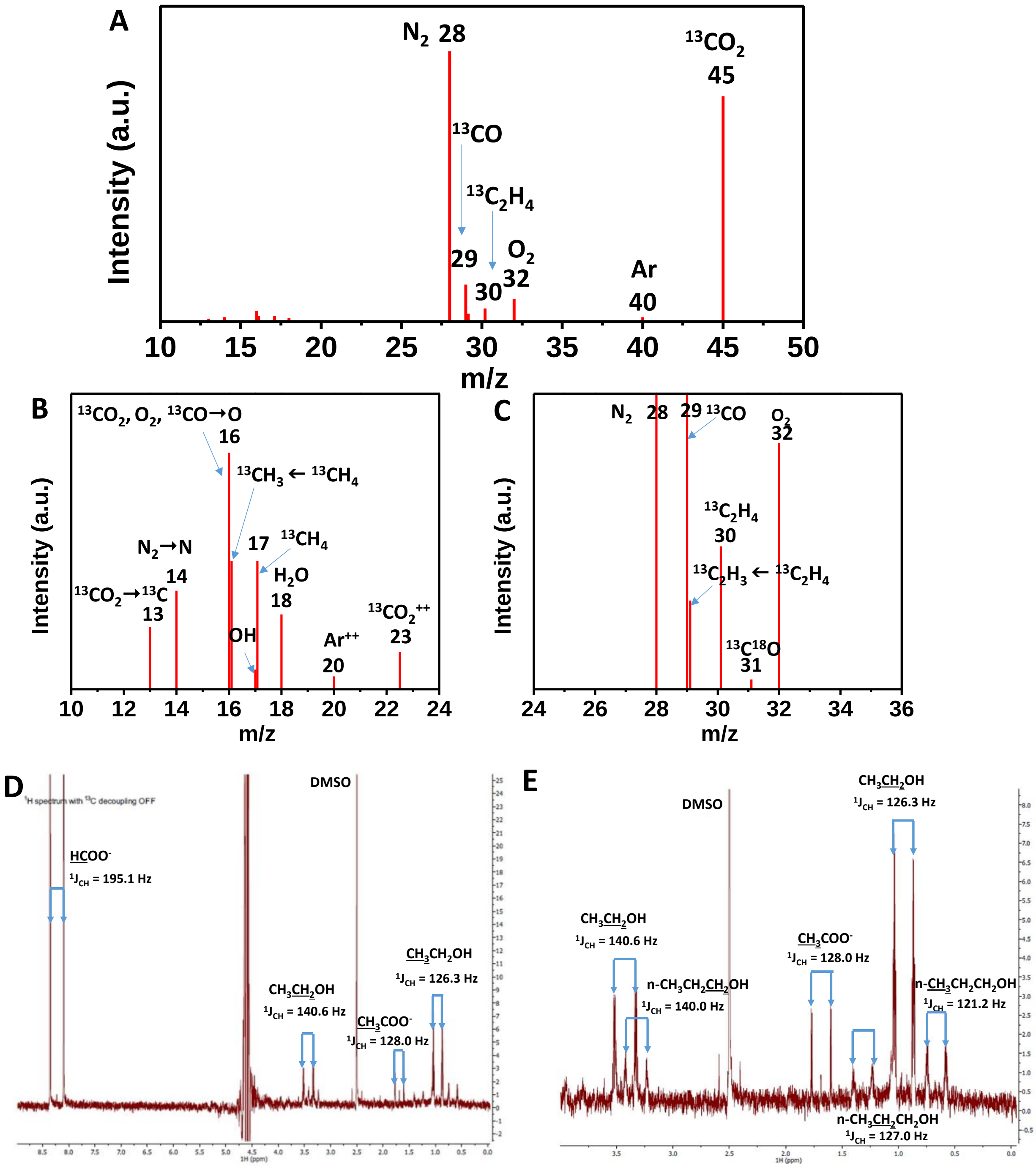

Supplementary Figure 7. Qualitative analysis of electrochemical reduction of isotopic $^{13}CO_2$. (A) Mass spectra of gas products. (B) and (C) zoom in of (A) in different m/z regions. (D) NMR of ^{1}H showing spin split when coupling to ^{13}C. (E) Zoom in of (D). A small percentages of ^{12}C contained products were also obtained as evidenced by a tiny ^{1}H NMR peak in between the two coupling ^{1}H NMR peaks in the products of $HCOO^-$ and CH_3COO^- because some $^{12}CO_2$ involves in the $^{13}CO_2$ gas.

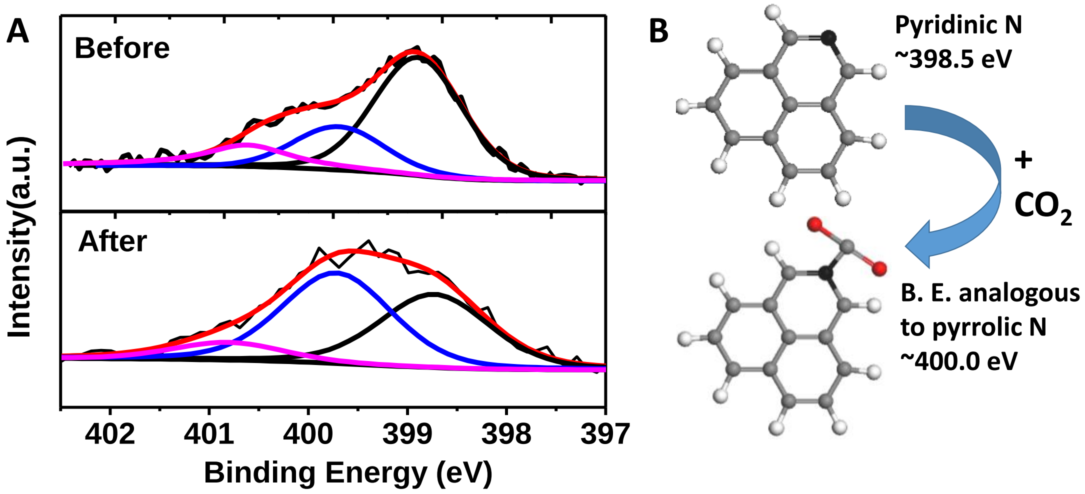

Supplementary Figure 8. The active N site for CO_2 adsorption. (A) Post-CO_2 reduction XPS analysis showing the change of N configuration concentration. (B) A schematic of CO_2 adsorption onto the pyridinic N site.

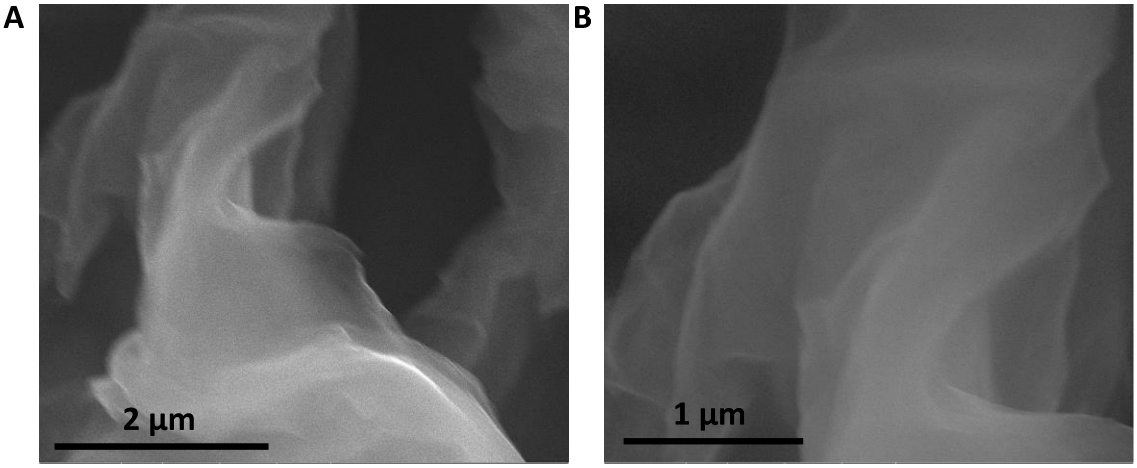

Supplementary Figure 9. SEM images of NRGOs. (A) Low-magnification images and (B) relatively high-magnification images.

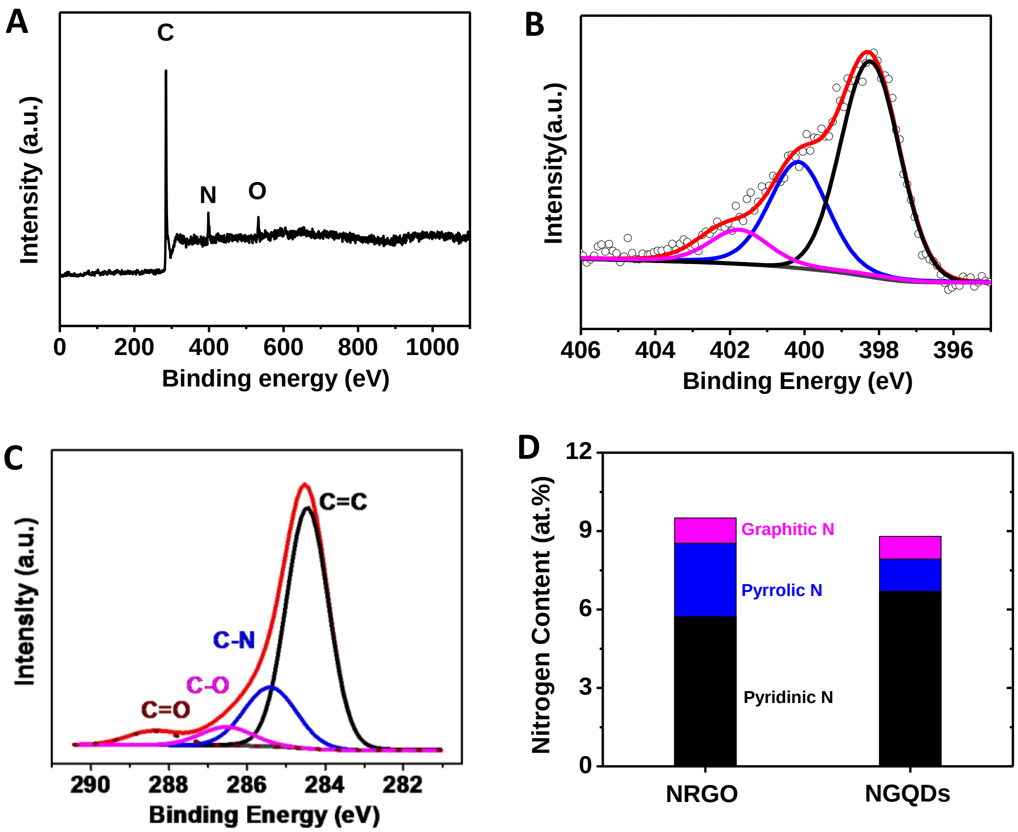

Supplementary Figure 10. XPS analysis of NRGOs. (A) Survey scan. (B) N 1s spectrum and its corresponding fitting. (C) C 1s spectrum and its deconvolution. (D) Comparison of specific N concentration between NRGOs and NGQDs.

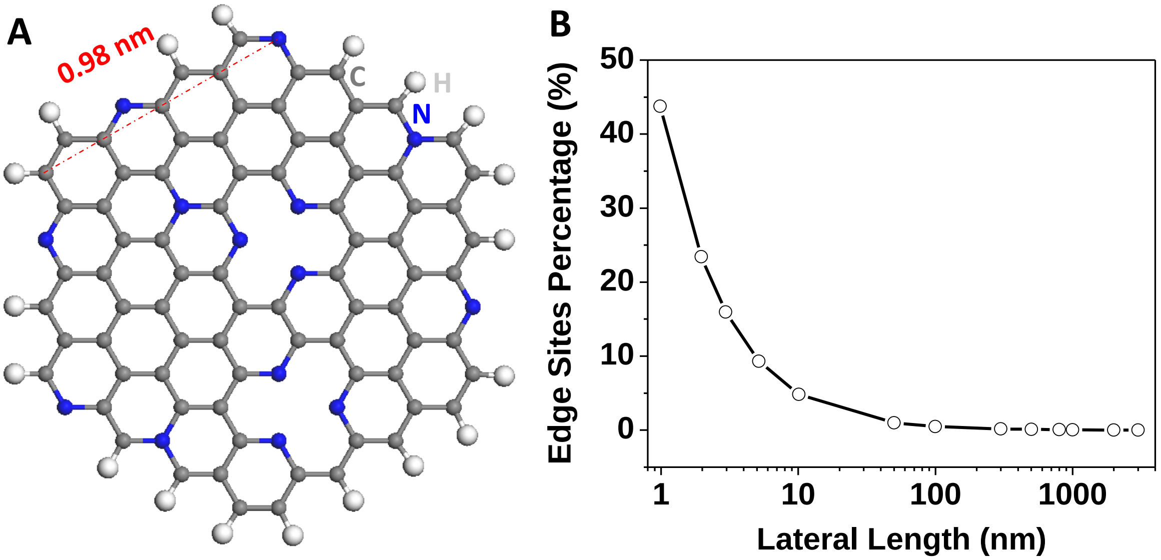

Supplementary Figure 11. **The fraction of edge site.** (A) Schematic of a NGQD with an edge length of 0.98 nm. The blue balls represent the N dopants. (B) Dependence of edge sites concentration for possible N doping on the edge length. The calculation is based on an ideal hexagonal NGQD shape. The equation used for calculation is: edge sites% = $(2n-1)/n^2$, n is the number of C6 aromatic ring in one edge of the hexagonal NGQD shape.

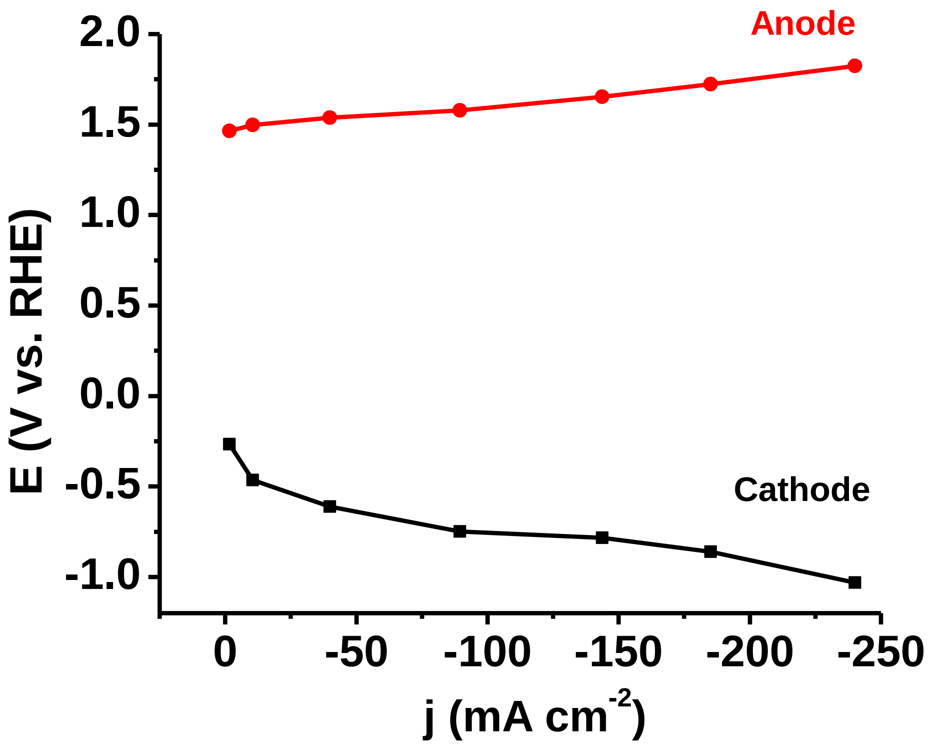

Supplementary Figure 12. iR corrected polarization curves for the flow electrolysis cell incorporating the NGQDs gas diffusion electrode.

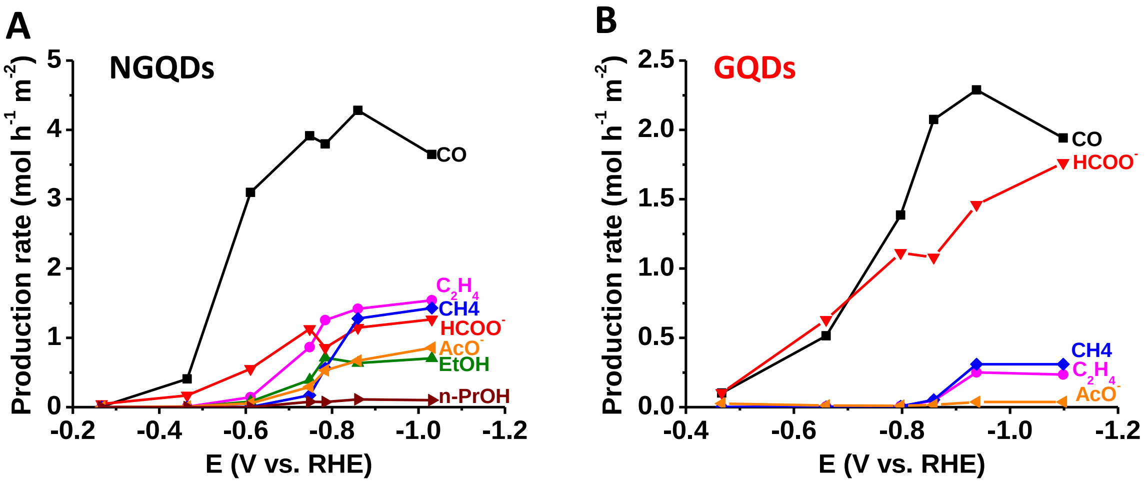

Supplementary Figure 13. Production rates of various products as a function of cathodic potentials from CO_2 reduction catalyzed using (A) NGQDs and (B) GQDs as the cathode catalyst.

Supplementary Methods

Graphene quantum dots synthesis

Improved Hummer's method was used to synthesize graphene oxide (GO) from SP1 Graphite powder. The procedure involves mixing 3 g of graphite powder with 18 g of $KMnO_4$ and 3 g $NaNO_3$ followed by slow addition of 360 mL H_2SO_4. It was then kept under stirring for 12 hours. After that the solution was poured onto ice (made from 500 mL DI water) and 14 mL H_2O_2 was added carefully. After stirring for an hour, the solution was allowed to stand for a day. The yellowish brown slurry that settled down was collected and the procedure for GO synthesis was again repeated on the collected product for further increasing the oxidation degree of GO, which is very important for controlling the final size of graphene quantum dots (GQDs). The collected slurry after the second oxidation step having a volume of ~300 mL, was then washed using 30% HCl, ethanol and DI water in sequence to remove any impurities. 500 mL 30% HCl was added into the 300 mL slurry, and stirred magnetically for 5 min under 1000 rpm. The subsidence was collected after centrifuging. The washing with HCl was repeated three times. After HCl washing, the subsidence was added with 500 mL ethanol, stirred for 5 min under 1000 rpm, and collected after centrifuging. The washing with ethanol was repeated three times. At last, the sample was washed with 500 mL DI water for three times. After first round DI water washing, the conductivity of 0.2 mg/mL GO solution ranged 50-100 µs/cm. Repeat the DI water washing process until the conductivity decreases down to 1.600 µs/cm, which is very close to the 1.014 µs/cm of DI water. Moreover, after washing, the trace metals concentration is ultra-low, like Na (0.06 at%) and Mn (0.05 at%) while no K is detected as shown in the XPS analysis.

The resulted GO was used as the precursor for hydrothermal alike synthesis of N-doped and pristine graphene quantum dots (GQDs). In the case of N-doped GQDs, typically 300 mg GO was dispersed in 30 ml dimethylformamide (DMF) and then sonicated in bath ultrasonicator for 30 min.[1] Afterwards, the GO suspension was transferred to a 50 ml PTFE liner. The NGQDs was formed in a hydrothermally analogous process at 200 °C for 10 h during which GO was exfoliated and cut at the weak sites with oxygen containing groups, and simultaneously doped by N into the carbon lattice with N source from DMF and its derived produce of dimethylamine, methylamine and ammonia. The pristine GQDs were synthesized using the same GO precursors and process except replacing DMF by a mixture of IPA and H_2O (1:1 by volume). The ratio is optimized to match the surface energy component of IPA/H_2O co-solvent to that of GO, so that to maximize the exfoliation and cutting efficiency.[2, 3] The N-doped reduced graphene oxide was prepared in a tube furnace at 800 °C while flowing ammonia for 1 h.

Gas diffusion electrode preparation

The cathodes were prepared using an air-brush method as previously reported.[4] Cathode catalyst inks for QDs were prepared by firstly mixing QDs solution (10 ml) and Nafion® solution (26 μL, 5 wt%, Fuel Cell Earth), and then being sonicated for 5 min. The cathode ink for NRGOs were prepared in the same manner except using NRGOs powder of 5 mg. Afterwards, the catalyst ink was air-brushed onto a gas diffusion layer (GDL, Sigracet 35 BC, Ion Power) to create a gas diffusion electrode (GDE). The catalyst loading for all cathode GDEs were kept at 0.5 ± 0.1 mg cm^{-2}. The anodes were

prepared by hand-painting of IrO_2 catalyst inks onto GDL to reach a loading of about 1.5 mg cm^{-2}.

<u>Electrochemical measurement</u>

An electrochemical flow cell composed of targeted GDE cathode and IrO_2 GDE anode as shown in Fig. S4 was employed to carry out CO_2 reduction at ambient pressure and temperature.[5, 6] The electrolysis was performed under potentiostatic mode with a full cell voltage ranging from −1.6 V to −3.5 V controlled by a potentiostat (Autolab PGSTAT-30, EcoChemie). Both the catholyte and anolyte were 1 M KOH (pH = 13.48, as calibrated by a pH meter (Thermo Orion, 9106BNWP). High purity CO_2 was supplied to the cathode at flowing rate of 7 SCCM monitored by a mass flow controller (MASS-FLO®, MKS instrument). The electrolyte was fed by a syringe pump (PHD 2000, Harvard Apparatus) with a continuing flowing to minimize boundary layer depletion effects and maintain the pH on the electrode surface with a supply of fresh electrolyte. The flow rate was set at 0.5 mL min^{-1} when applying cell potentials more negative than -2 V, otherwise using a slower flow rate of 0.1 mL min^{-1} to increase the concentration of the liquid products at a relative lower current density.

For each applied voltage, after the cell reached steady state, 1 mL of the effluent gas stream was periodically sampled and diverted into a gas chromatograph (Thermo Finnegan Trace GC) equipped with both the thermal conductivity detection (TCD) and flame ionization detector (FID), and a Carboxen 1000 column (Supelco). Three successive injections of effluent gas stream with 1.6 mins interval between each injection were directed into the GC wherein the second and third injection occurred before the first was allowed to elute to save the overall running time. The gas peaks for respective

injection are separated as shown in Figure S5A. Helium as the carrier gas flows at a rate of 20 SCCM. Meanwhile, the exit catholyte was collected at each applied voltage followed by identifying and quantifying using ^{1}H NMR (nuclear magnetic resonance, UI500NB, Varian). 100 μL of the catholyte was mixed with 100 μL internal standard of 1.25 mM DMSO (99.98%, Calbiochem) in 400 μL D_2O (99.9% deuterium atom, Sigma-Aldrich). The current reported here was obtained by averaging the span of time (at least 180 s) for each applied voltage.

Individual electrode potentials were recorded using multimeters (AMPROBE 15XP-B) connected to each electrode and a reference electrode (Ag/AgCl; RE-5B, BASi) placed in the electrolyte exit stream. The measured potentials after iR compensation were rescaled to the RHE by E (vs. RHE) = E (vs. Ag/AgCl) + 0.209 V + 0.0591 V/pH × pH.

The onset potential is defined as the lowest cathode potential at which product was detected from either GC or NMR. The gas products from $^{13}CO_2$ were identified by VG 70S double-focusing magnetic sector mass spectrometer.

The Faradaic efficiency (FE) for a specific product is calculated using the following equation:

$FE = (z \times n \times F)/Q$

where

z = the number of electrons exchanged (for example, z = 2 for reduction of CO_2 to CO),

n = the number of moles for a specific product,

F = Faraday's constant (F = 96485 C/mol),

Q = the charge passed (C).

Supplementary References:

1. Sun, J., Yang S., Wang Z., Shen H., Xu T., Sun L., *et al.* Ultra-High Quantum Yield of Graphene Quantum Dots: Aromatic-Nitrogen Doping and Photoluminescence Mechanism. *Part. Part. Syst. Charact.* **32,** 434-440 (2015).

2. Shen, J., He Y., Wu J., Gao C., Keyshar K., Zhang X., *et al.* Liquid Phase Exfoliation of Two-Dimensional Materials by Directly Probing and Matching Surface Tension Components. *Nano Lett.* **15,** 5449-5454 (2015).

3. Shen, J., Wu J., Wang M., Dong P., Xu J., Li X., *et al.* Surface Tension Components Based Selection of Cosolvents for Efficient Liquid Phase Exfoliation of 2D Materials. *Small* **12,** 2741-2749 (2016).

4. Jhong, H.-R. M., Brushett F. R., Kenis P. J. A. Fuel Cells: The Effects of Catalyst Layer Deposition Methodology on Electrode Performance *Adv. Energy Mater.* **3,** 541-541 (2013).

5. Ma, S., Sadakiyo M., Luo R., Heima M., Yamauchi M., Kenis P. J. A. One-step electrosynthesis of ethylene and ethanol from CO_2 in an alkaline electrolyzer. *J. Power Sources* **301,** 219-228 (2016).

6. Ma, S., Luo R., Moniri S., Lan Y., Kenis P. J. A. Efficient Electrochemical Flow System with Improved Anode for the Conversion of CO_2 to CO. *J. Electrochem. Soc.* **161,** F1124-F1131 (2014).
